# Spatiotemporal dynamics and quantitative analysis of phytoplasmas in insect vectors

**DOI:** 10.1038/s41598-020-61042-x

**Published:** 2020-03-09

**Authors:** Hiroaki Koinuma, Kensaku Maejima, Ryosuke Tokuda, Yugo Kitazawa, Takamichi Nijo, Wei Wei, Kohei Kumita, Akio Miyazaki, Shigetou Namba, Yasuyuki Yamaji

**Affiliations:** 1 Graduate School of Agricultural and Life Sciences, The University of Tokyo, 1-1-1 Yayoi, Bunkyo-ku, Tokyo 113-8657 Japan; 2 Molecular Plant Pathology Laboratory, US Department of Agriculture–Agricultural Research Service, Beltsville, MD 20705 USA

**Keywords:** Cellular microbiology, Entomology

## Abstract

Phytoplasmas are transmitted by insect vectors in a persistent propagative manner; however, detailed movements and multiplication patterns of phytoplasmas within vectors remain elusive. In this study, spatiotemporal dynamics of onion yellows (OY) phytoplasma in its vector *Macrosteles striifrons* were investigated by immunohistochemistry-based 3D imaging, whole-mount fluorescence staining, and real-time quantitative PCR. The results indicated that OY phytoplasmas entered the anterior midgut epithelium by seven days after acquisition start (daas), then moved to visceral muscles surrounding the midgut and to the hemocoel at 14–21 daas; finally, OY phytoplasmas entered into type III cells of salivary glands at 21–28 daas. The anterior midgut of the alimentary canal and type III cells of salivary glands were identified as the major sites of OY phytoplasma infection. Fluorescence staining further revealed that OY phytoplasmas spread along the actin-based muscle fibers of visceral muscles and accumulated on the surfaces of salivary gland cells. This accumulation would be important for phytoplasma invasion into salivary glands, and thus for successful insect transmission. This study demonstrates the spatiotemporal dynamics of phytoplasmas in insect vectors. The findings from this study will aid in understanding of the underlying mechanism of insect-borne plant pathogen transmission.

## Introduction

Many plant pathogens of agricultural importance are persistently transmitted by insect vectors^1,2^. The circulation of these pathogens within insect vectors involves several steps: (i) entry into and multiplication in gut epithelial cells of the alimentary canal, (ii) entry into the hemocoel and circulation in hemolymph, and (iii) final entry into and multiplication in salivary glands, which results in successful pathogen transmission to new plants *via* insect saliva^2–4^. Previous studies of plant viruses and plant pathogenic bacteria have shown that the alimentary canal and salivary glands are the major organs that determine vector competence^3–5^. Thus, the timing and location of insect-borne plant pathogen infection and passage through the target organs is fundamental to the understanding of underlying infection mechanisms. The infection site^6,7^ and temporal localization^8^ of spiroplasmas (*Spiroplasma citri* and *S. kunkelii*) have been revealed by transmission electron microscopy (TEM) studies of infected alimentary canal and salivary glands of leafhoppers (*Circulifer tenellus* and *Dalbulus maidis*). In addition, the localizations of spiroplasma (*S. kunkelii*)^9^ and liberibacter (‘*Candidatus* Liberibacter asiaticus’)^10^ in their vector organs have also been visualized by fluorescence microscopic studies. However, spatial and temporal distribution patterns of these bacterial pathogens at the organ level remain elusive.

Phytoplasmas are cell wall-less plant pathogenic bacteria that, together with spiroplasmas, belong to the class *Mollicutes*. They infect more than 1,000 plant species and cause severe losses in the yields of many important crops worldwide^11,12^. Phytoplasmas inhabit the phloem of host plants and are transmitted among plants in a persistent propagative manner by phloem-sucking insects, such as leafhoppers, planthoppers, and psyllids^2,5^. A prior experiment involving injection of phytoplasma crude extracts prepared from dissected internal organs to phytoplasma-free insect vectors revealed that phytoplasmas circulated sequentially in the alimentary canal, hemolymph, and salivary glands^13^, which is similar to the localizations of other persistently transmitted pathogens.

The temporal localizations of clover phyllody and X-disease phytoplasmas have been observed in leafhoppers by TEM^14–16^. Serology- and molecular biology-based detection methods, such as enzyme-linked immunosorbent assay (ELISA)^17^ and polymerase chain reaction (PCR)^18^, have also been used to examine the temporal localizations of flavescence dorée (FD) and onion yellows (OY) phytoplasmas. However, these methods have their limitations. TEM-based high-magnification approach is suitable for observation of phytoplasmas at the cellular and subcellular levels, but not for visualization at the whole-body, organ, or tissue levels. Moreover, TEM cannot readily distinguish among different phytoplasmas, or phytoplasmas from other cell wall-less bacteria, when mixed infections occur^19,20^. ELISA- and PCR-based analyses can detect and quantify specific phytoplasmas at the whole-body, organ, or tissue levels^17,18,20–24^; however, they cannot determine whether phytoplasmas are located inside or outside the organs. Because these methods have been used independently in most studies, a detailed understanding is lacking with respect to the movements and multiplication patterns of phytoplasmas in insect vectors, particularly the timing and locations of phytoplasma passage through various insect organs, such as the alimentary canal and salivary glands; most importantly, there remains a lack of clarity regarding the duration and abundance of phytoplasmas accumulated in each organ.

In the present study, we combined immunohistochemistry-based three-dimensional (3D) imaging technology, whole-mount immunofluorescence techniques and real-time quantitative PCR (qPCR) to examine the spatiotemporal dynamics and accumulation patterns of OY phytoplasmas in the whole-body and internal organs of its insect vector, *Macrosteles striifrons*. An immunofluorescence analysis using whole-mount organs enabled us to distinguish between phytoplasmas located inside and outside of the organs. qPCR analysis revealed the spatiotemporal relationship between phytoplasma accumulation and distribution in insect vectors. Our findings from this study provide information regarding the underlying mechanism of insect-borne plant pathogen transmission.

## Results

### Spatiotemporal distribution of OY phytoplasmas in entire insect vector based on 3D imaging technology

Our previous studies have successfully detected OY phytoplasmas in insect vectors by immunohistochemical staining, using an antibody raised against an immunodominant (antigenic) membrane protein (Amp) of OY phytoplasma^25–27^. The anti-Amp antibody has been shown not to react to the sections of negative control insects^26^. Therefore, in the present study, single paraffin sections were not used; in contrast, the entire insect body was embedded in paraffin, serially sectioned, and immunohistochemically stained using the anti-Amp antibody as the primary antibody, along with an alkaline-phosphatase-conjugated secondary antibody, to enable processing and reconstruction of 3D images. Using these 3D images, the spatiotemporal distribution of OY phytoplasmas in entire insect vectors at different days after acquisition start (daas) was investigated. Insects were reared on diseased plants for 7 days for acquisition feeding; transferred to healthy plants; collected at 1, 2, 4, 6, 10, 14, 20, 27, 34, and 41 daas; and processed for immunohistochemical analysis.

**Figure 1:**
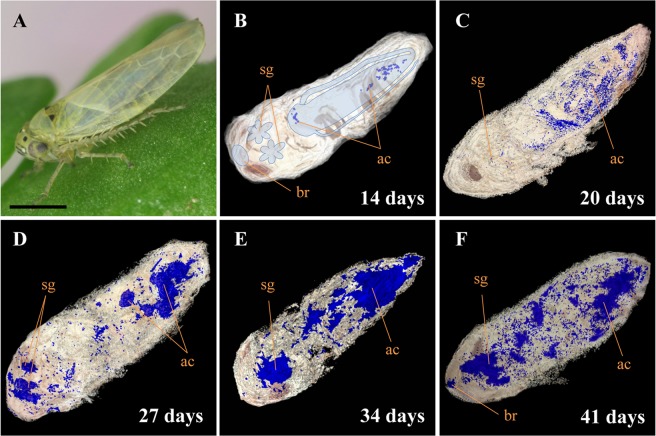
Distribution of onion yellows (OY) phytoplasmas in leafhopper vector *Macrosteles striifrons* by three-dimensional (3D) imaging technology. (**A**) *Macrosteles striifrons* vector. (**B–F**) 3D visualization of OY phytoplasmas (blue signals) in its vector, collected at 14 (**B**), 20 (**C**), 27 (**D**), 34 (**E**), and 41 (**F**) days after acquisition start (daas) on diseased plants, based on immunohistochemical staining using anti-Amp primary antibody and alkaline-phosphatase-conjugated secondary antibody. The alimentary canal, salivary glands, and brain of the insect are outlined in panel B. ac, alimentary canal; sg, salivary gland; br, brain. Bar, 1 mm.

In leafhopper, the alimentary canal occupies most of the abdomen, while salivary glands are located between the thorax and head. The brain is located at the tip of the head^28,29^. Amp-specific blue signals of OY phytoplasma were first detected in the alimentary canal (Fig. 1B) at 14 daas, and then observed throughout the abdomen, including in the alimentary canal and a portion of the salivary glands at 20 daas (Fig. 1C). Thereafter, stronger signals were identified in the alimentary canal and salivary glands at 27 and 34 daas (Fig. 1D,E). At 41 daas, signals were also found in the brain (Fig. 1F). Taken together, the results of this study indicated that OY phytoplasmas moved within *M. striifrons* from the alimentary canal to the hemocoel, then to the salivary glands, and finally to the brain. The alimentary canal and the salivary glands are major internal organs for phytoplasma multiplication; abundant accumulation of OY phytoplasmas began at 27 daas.

### Quantitative analysis of OY phytoplasma accumulation in insect vectors

OY phytoplasma accumulation at the whole-body and organ or tissue levels was quantified by qPCR. Because immunohistochemical staining has shown that OY phytoplasmas exit from the alimentary canal and move towards the salivary glands at 27 daas (Fig. 1), insects given a 7-day acquisition feeding period on a diseased plant, followed by transfer to a healthy plant, were collected at 7, 14, 21, and 28 daas for quantitative analysis. DNA was extracted from entire insect bodies, dissected alimentary canals, legs (which represent hemocoel^30^), and salivary glands. The results are summarized in Supplementary Table S1. No amplification was observed in negative control samples.

**Figure 2:**
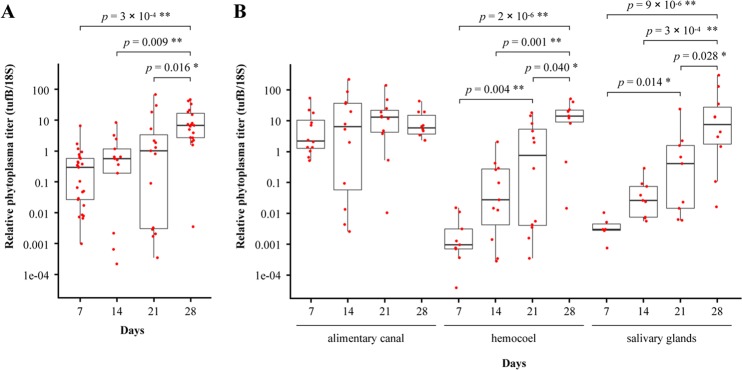
Time course analysis of OY phytoplasma accumulation in *M. striifrons* by qPCR. Accumulations of OY phytoplasmas in the entire insect body (**A**) and in the alimentary canal, hemocoel, and salivary glands (**B**) were quantified at different days after acquisition start by qPCR. Only OY phytoplasma-positive samples were used to measure the relative phytoplasma titer. Each red point represents an OY-positive sample. Four dissected organs/tissues were pooled as one sample. Thick horizontal line in each boxplot represents the median of each dataset. Statistical significance was determined using Tukey’s honestly significant difference test. Only significant comparisons are shown. *p < 0.05, **p < 0.01.

qPCR revealed that phytoplasma titer increased gradually from 7 to 21 daas, and was significantly elevated at 28 daas throughout the insect (Fig. 2A). In dissected alimentary canals, phytoplasma multiplied approximately 5-fold between 7 and 21 daas, and then showed no significant change from 21 to 28 daas (Fig. 2B). At 7 daas, the phytoplasma titer was extremely low or below the detection limit in the hemocoel and salivary glands (Supplementary Table S1, Fig. 2B). However, from 7 to 28 daas, phytoplasma began accumulating at a fast pace; titer then increased approximately 10-fold per week, finally reaching levels comparable to those in the alimentary canal at 28 daas (Fig. 2B).

### Visualization of OY phytoplasmas in the alimentary canal and the salivary glands by whole-mount immunofluorescence staining

**Figure 3:**
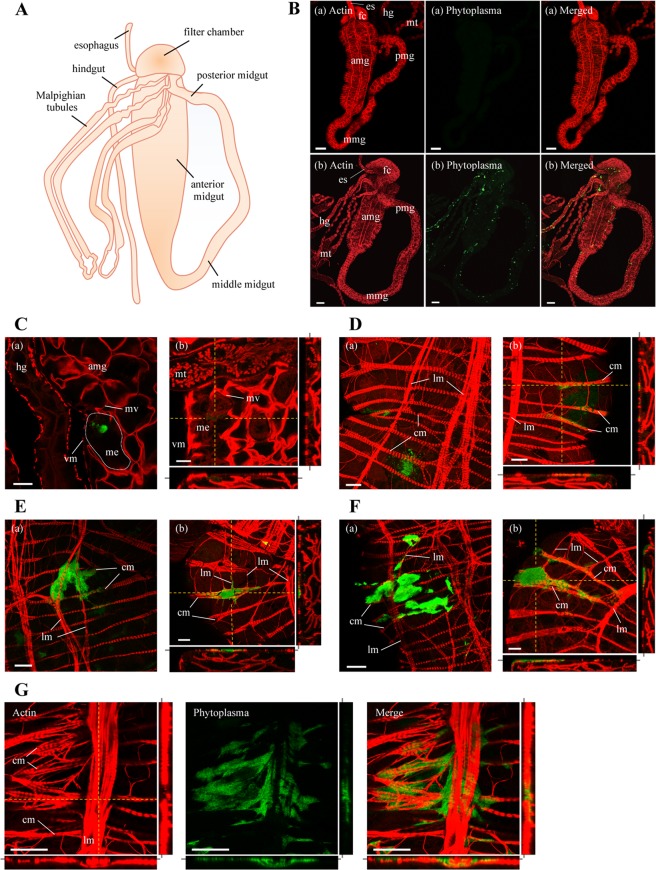
Distribution of OY phytoplasmas in the alimentary canal of *M. striifrons*. (**A**) Diagrammatic outline of the alimentary canal of *M. striifrons*. (**B–G**) Whole-mount immunofluorescence staining of the alimentary canal using Amp-Alexa Fluor 488 (green) and the actin dye phalloidin-Alexa Fluor 546 (red). (**B**) Alimentary canals of a non-infected leafhopper (a) and an OY-infected leafhopper that was consistently maintained on a diseased plant for more than 1 month (b). (**C–G**) Luminal side of anterior midguts of OY-infected leafhoppers at 7 daas (**C**) and muscle side at 14 (**D**), 21 (**E**), and 28 (**F,G**) daas. Images (a,b) in panels C–F are two representative images from the same daas. Image b in panels C–F, and images in panel G, were obtained along the *xy* plane (square photos) or along *yz* and *xz* planes (vertical and horizontal rectangular photos, respectively). Rectangular photos were reconstructed from Z-stacks of confocal images, indicated by yellow dashed vertical and horizontal lines in square photos. Gray lines extending from rectangular photos indicate positions of square photos from Z-stacks. All images are representative of more than three experiments. es, esophagus; fc, filter chamber; amg, anterior midgut; mmg, middle midgut; pmg, posterior midgut; hg, hindgut; mt, Malpighian tubules; me, midgut epithelium; mv, microvilli; vm, visceral muscle; cm, circular muscle; lm, longitudinal muscle. Bar, 100 µm (**B**), 25 µm (**C–G**).

**Figure 4:**
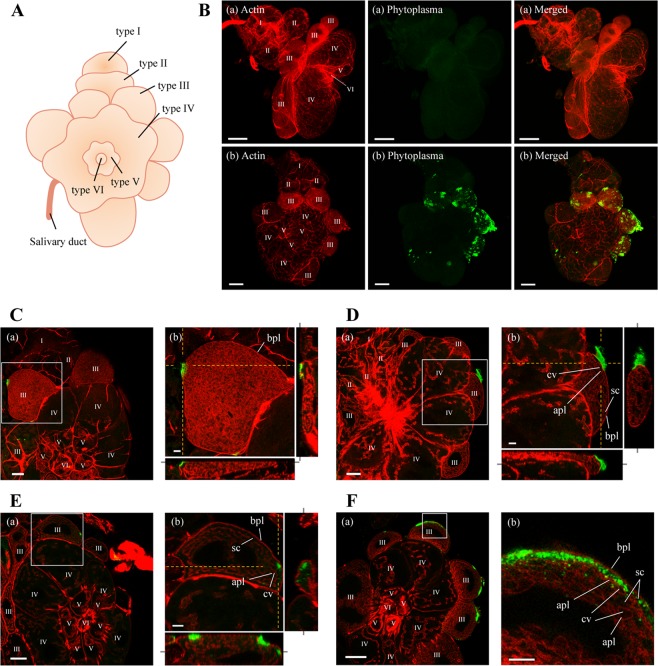
Distribution of OY phytoplasmas in salivary glands of *M. striifrons*. (**A**) Diagrammatic outline of the principal salivary gland of *M. striifrons*. (**B–F**) Whole-mount immunofluorescence staining of salivary glands using Amp-Alexa Fluor 488 (green) and the actin dye phalloidin-Alexa Fluor 546 (red). (**B**) Salivary glands of a non-infected leafhopper (a) and an OY-infected leafhopper that was consistently maintained on a diseased plant for more than 1 month (b). (**C–F**) Salivary glands of OY-infected leafhoppers at 14 (**C**), 21 (**D**), and 28 (**E,F**) daas. Images in panels C–F are magnified images of boxed areas in image a in the same panel. Image b in each of the panels (**C–E**) was obtained as described in Fig. 3. All images are representative of more than three experiments. I–VI refers to type I–VI cells; bpl, basal plasmalemma; sc, salivary cytoplasm; apl, apical plasmalemma; cv, cavity. Bar, 75 µm (image a in **B**), 50 µm (image b in **B**, image a in **F**), 25 µm (image a in **C–E**), 10 µm (image b in **C–F**).

In addition to the spatiotemporal distribution of OY phytoplasmas at the whole-body level by immunohistochemistry-based 3D visualization, the spatiotemporal localizations of OY phytoplasmas in the alimentary canal and salivary glands at the organ level were investigated by whole-mount immunofluorescence staining combined with confocal microscopy. The alimentary canal of a leafhopper contains four parts, including the esophagus (foregut), filter chamber, midgut, and hindgut. The midgut consists of three major regions: anterior, middle, and posterior midgut (Fig. 3A)^29^. The salivary glands of leafhoppers consist of one set each of the principal and accessory glands; the principal glands are divided into six cell types (I–VI) (Fig. 4A)^31^. In positive control samples, Amp-specific green fluorescence of OY phytoplasmas was observed in all parts of the alimentary canals and type I–IV cells of salivary glands, whereas no fluorescence (auto-fluorescence) was observed in negative controls (Figs. 3B and 4B). This finding indicated that whole-mount immunofluorescence staining was feasible for visualization of the localizations of OY phytoplasmas in leafhoppers. Therefore, insects given a 7-day acquisition feeding period followed by a transfer to a healthy plant were collected at different daas and processed for whole-mount immunofluorescence staining.

### Movement and accumulation patterns of OY phytoplasmas in the alimentary canal

By using immunofluorescence staining, OY phytoplasma-specific green fluorescence was detected in 13 of 20 (65%), 12 of 20 (60%), 13 of 20 (65%), and 22 of 28 (79%) alimentary canal samples collected at 7, 14, 21, and 28 daas, respectively (Table 1). In addition, OY phytoplasma distribution was examined in different regions of the alimentary canal, including the filter chamber, anterior midgut (i.e., epithelium, limited area of visceral muscles, and extensive area of visceral muscles), middle midgut, posterior midgut, hindgut, and Malpighian tubules. At 7 daas, OY-specific fluorescence was observed in three regions of the alimentary canals: filter chamber, epithelium of anterior midgut, and middle midgut. At 14 daas, phytoplasmas were also observed in four other regions: limited area of visceral muscles of anterior midgut, posterior midgut, hindgut, and Malpighian tubules. Subsequently, phytoplasmas were detected in all tested regions of the alimentary canal at both 21 and 28 daas; in addition, the detection positive rate in tested samples increased from 21 to 28 daas. Importantly, OY phytoplasmas were most frequently located in the epithelium of anterior midgut, compared to other regions.

**Table 1:** Distribution of OY phytoplasma in tissues of *M. striifrons*, as detected by immunofluorescence microscopy.

Tissues examined	No. of positive tissues of OY Amp antigens at different daas^a^							
	7 days		14 days		21 days		28 days	
	No. detected / no. tested	ratio (%)	No. detected / no. tested	ratio (%)	No. detected / no. tested	ratio (%)	No. detected / no. tested	ratio (%)
Alimentary canal	13/20	65	12/20	60	13/20	65	22/28	79
Filter chamber	7/20	35	6/20	30	11/20	55	16/28	57
Anterior midgut (epithelium)	11/20	55	12/20	60	12/20	60	21/28	75
Anterior midgut (limited area of visceral muscles)	0/20	0	4/20	20	11/20	55	20/28	71
Anterior midgut (extensive area of visceral muscles)	0/20	0	0/20	0	4/20	20	13/28	46
Middle midgut	1/20	5	4/20	20	6/20	30	13/28	46
Posterior midgut	0/20	0	2/20	10	5/20	25	15/28	54
Hindgut	0/20	0	3/20	15	3/20	15	11/28	39
Malpighian tubules	0/20	0	1/20	5	8/20	40	15/28	54
Salivary glands	0/20	0	4/20	20	12/20	60	20/28	71
I cell	0/20	0	1/20	5	3/20	15	7/28	25
II cell	0/20	0	3/20	15	4/20	20	9/28	32
III cell (surface)	0/20	0	4/20	20	12/20	60	20/28	71
III cell (inside)	0/20	0	0/20	0	3/20	15	9/28	32
IV cell	0/20	0	2/20	10	5/20	25	16/28	57
V cell	0/20	0	2/20	10	4/20	20	10/28	36
VI cell	0/20	0	0/20	0	1/20	5	4/28	14

^a^daas: days after acquisition start on the diseased plants.

The midgut consists of a single layer of epithelial cells with microvilli on the luminal side and a basal lamina on the outer side^32^. It is surrounded by visceral muscles made up of actin-based internal circular muscle fibers and external longitudinal muscle fibers^32^. At 7 daas, Amp-specific fluorescence of OY phytoplasmas was observed in the epithelium of anterior midgut (Table 1, Fig. 3C, Supplementary Video S1), but not in the visceral muscles (Table 1, Supplementary Fig. S1A, Supplementary Video S1). Beginning at 14 daas, fluorescence was observed in the gut epithelium (Table 1, Supplementary Fig. S1B–E), but was mainly concentrated in the visceral muscles (Table 1, Fig. 3D–F), indicating the movement of most phytoplasmas from the gut epithelium to visceral muscles at this point. In addition, fluorescence was occasionally observed along cytoplasmic actin in the gut epithelium (Supplementary Fig. S1E, Supplementary Video S3), suggesting intracellular movement of phytoplasmas along microfilaments. The high detection rate in the visceral muscles at 21 daas (Table 1) also suggested that OY phytoplasmas reached the visceral muscles by 21 daas.

In the visceral muscles, OY phytoplasma-specific fluorescence was first detected near the internal circular muscle fibers at 14 daas (Fig. 3D, Supplementary Video S2), in both the internal circular muscle fibers and external longitudinal muscle fibers at 21 daas (Fig. 3E, Supplementary Video S3), and extensively along these fibers at 28 daas (Table 1, Fig. 3F, Supplementary Video S4). The fluorescence appeared to spread along the internal circular muscle fibers to the external longitudinal muscle fibers (Fig. 3G). These results indicated the movement of OY phytoplasmas towards the basal lamina, with a portion passing through the basal lamina to reach the internal circular muscle fibers at 14 daas and the external longitudinal muscle fibers at 21 daas, before finally spreading along these fibers at 28 daas.

**Figure 5:**
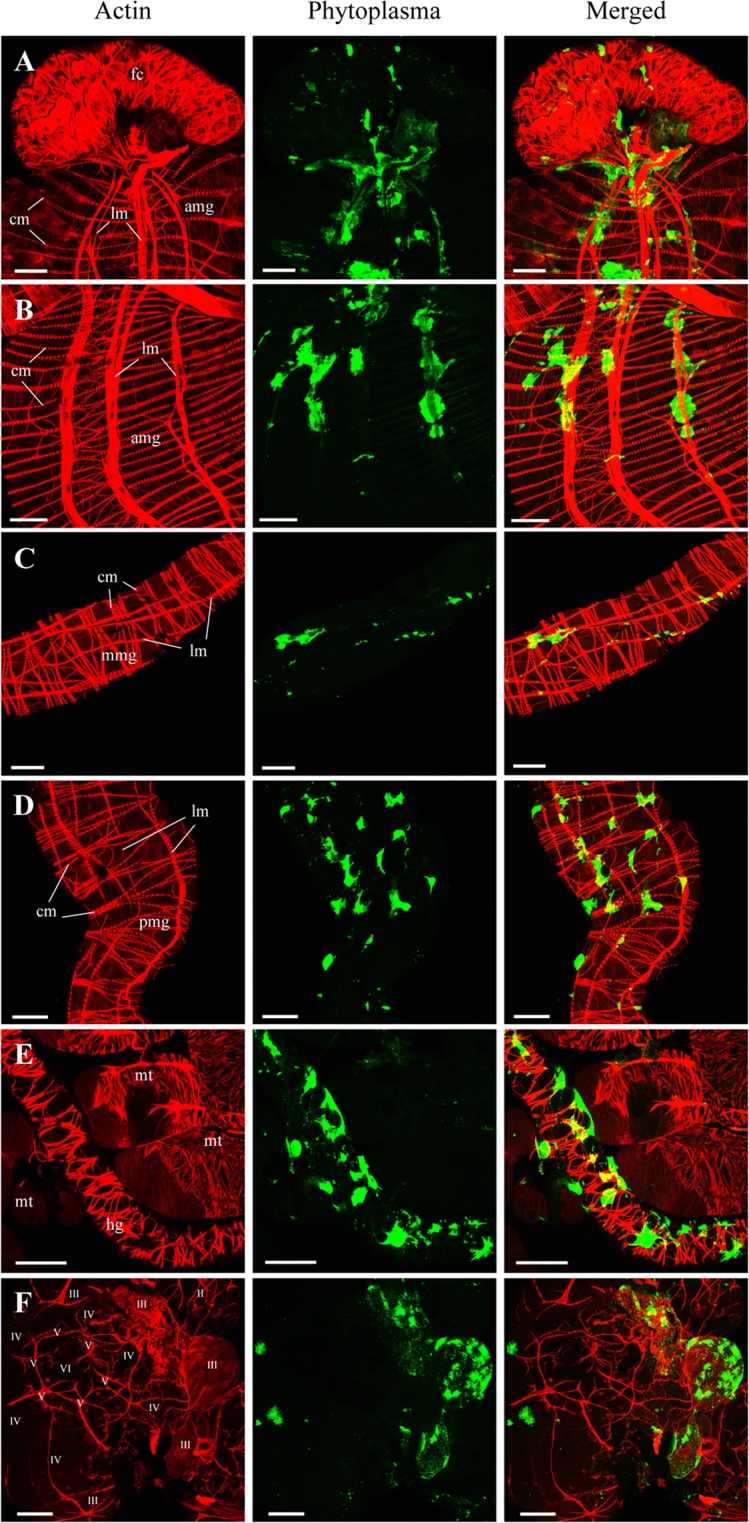
Distributions of OY phytoplasma in internal organs or tissues of *M. striifrons*. Whole-mount immunofluorescence staining of internal organs of OY-infected leafhoppers at 28 daas using Amp-Alexa Fluor 488 (green) and the actin dye phalloidin-Alexa Fluor 546 (red). Muscle side of the filter chamber (fc) (**A**), anterior midgut (amg) (**B**), middle midgut (mmg) (**C**), posterior midgut (pmg) (**D**), and hindgut (hg) and Malpighian tubules (mt) (**E**), as well as the outer side of the salivary gland (**F**), are shown. Images are representative of more than three experiments. cm, circular muscle; lm, longitudinal muscle; II–VI, type II–VI cells. Bar, 50 µm.

In addition, at 28 daas, fluorescence was observed in the visceral muscles, including the filter chamber, anterior midgut, middle midgut, posterior midgut, hindgut, and Malpighian tubules (Table 1, Fig. 5A–E). In contrast, fluorescence was not detected in the gut epithelium, except in the filter chamber and anterior midgut (Supplementary Fig. S2A–E). Thus, the localization of OY phytoplasmas at 28 daas was not restricted to the anterior midgut alone; it also had spread to other tissues of the alimentary canal. The abundant and intensive fluorescence in the visceral muscles (Figs. 3E–G and 5A–E) suggested vigorous multiplication of phytoplasmas at this site.

### Movement and accumulation patterns of OY phytoplasmas in salivary glands

Amp-specific fluorescence of OY phytoplasmas was detected beginning at 14 daas (Table 1). Only 20% (four of 20) samples were positive at 14 daas; the positive detection rate gradually increased at 21 and 28 daas. In type III cells of the salivary glands, intense fluorescence was frequently observed (Table 1).

Salivary gland cells are composed of a single layer of secretory cells, with apical plasmalemma-lined cavities (intracellular canaliculi) for saliva secretion and a basal plasmalemma covered by the basal lamina on the surface^6,31,33^. In type III cells, actin filaments are distributed on the inner sides of both basal plasmalemma (Fig. 4C–F) and apical plasmalemma^33^. OY Amp-specific fluorescence was observed on the surfaces of type III cells at 14 daas (Table 1, Fig. 4C, Supplementary Video S5). The high detection rate of OY Amp-specific fluorescence on the surfaces of type III cells at 21 daas (Table 1) suggested that OY phytoplasmas had reached type III cells by 21 daas. Although the fluorescence intensity on the cell surface increased at 21 daas, fluorescence was generally not observed within type III cells (Table 1, Fig. 4D, Supplementary Video S6). At 28 daas, OY Amp-specific fluorescence was occasionally present in the cytoplasm of type III cells (Table 1, Fig. 4E,F, Supplementary Fig. S2F, Supplementary Video S7) and in the space near the apical plasmalemma (Fig. 4F), indicating the invasion of OY phytoplasmas into type III cells. In addition, high-intensity fluorescence was observed in all salivary gland cell types, especially in type III cells, at 28 daas (Table 1, Fig. 5F).

## Discussion

In recent years, phytoplasma research has been flourishing in a variety of fields. Phytoplasmas are transmitted among plants by insect vectors in a persistent propagative manner. The mechanisms by which phytoplasmas circulate and accumulate within vectors and in internal organs or tissues are important for the understanding of vector-borne phytoplasma transmission. However, phytoplasma movement and accumulation patterns within insect vectors, as well as the timing of phytoplasma entries into and exits from these organs, remain unknown. In the present study, we investigated the spatiotemporal dynamics of OY phytoplasmas in its leafhopper vector, *M. striifrons*. This study shows that immunohistochemistry-based 3D imaging technology using a phytoplasma-specific antibody could visualize phytoplasma spread at the whole-body level (Fig. 1). qPCR and whole-mount immunofluorescence analysis demonstrated the accumulation and spatial and temporal distributions of OY phytoplasmas in the alimentary canal and salivary glands (Figs. 2B, 3–5), both of which are organs that determine vector competence^3–5^. The present study provided information on the movement and accumulation patterns of phytoplasmas during their passage through the vector.

**Figure 6:**
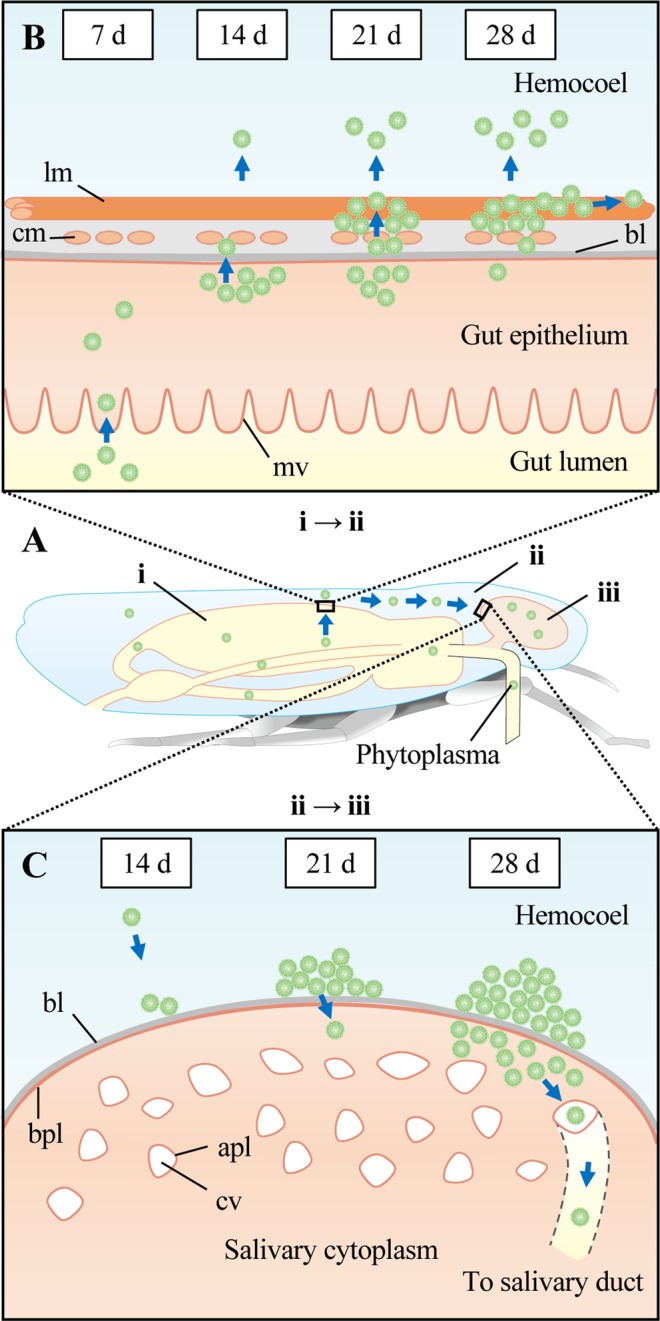
Proposed model of spatiotemporal dynamics of OY phytoplasma in its leafhopper vector, *M. striifrons*. (**A**) Dynamics of OY phytoplasmas in the entire insect body. OY phytoplasmas are acquired from plant phloem sap *via* the insect’s stylet. OY phytoplasmas enter the lumen of the alimentary canal (i), then pass through the canal into the hemocoel (i–ii). After circulating in the hemolymph, OY phytoplasmas finally reach the salivary glands (ii–iii). (**B**) Dynamics of OY phytoplasmas in the alimentary canal. By 7 days after acquisition start (daas; indicated as “d” in this figure), phytoplasmas enter the gut epithelium from the gut lumen through microvilli. At 14 daas, phytoplasmas multiply in the gut epithelium and move towards the basal lamina. Some phytoplasmas pass through the basal lamina to the internal circular muscle fibers and the hemocoel (i–ii). By 21 daas, many phytoplasmas cross the basal lamina to colonize both the internal circular muscle and external longitudinal muscle fibers and enter the hemocoel. At 28 daas, phytoplasmas mainly colonize the visceral muscles, spreading along muscle fibers. (**C**) Dynamics of OY phytoplasmas in salivary glands. A few phytoplasmas arrive at the basal lamina of the salivary gland cell at 14 daas (ii–iii). By 21 daas, many phytoplasmas accumulate on the surfaces of salivary gland cells; by 28 daas, some of these phytoplasmas enter into the cytoplasm. Thereafter, phytoplasmas in the cytoplasm pass through the apical plasmalemma of the salivary cavity and move into the salivary duct. mv, microvilli; bl, basal lamina; cm, circular muscle; lm, longitudinal muscle; bpl, basal plasmalemma; apl, apical plasmalemma; cv, cavity.

Based on the results of this study, we propose a model of the spatiotemporal dynamics of OY phytoplasmas in its vector (Fig. 6): OY phytoplasma enters the anterior midgut epithelium of the alimentary canal through microvilli by 7 daas and multiplies in the gut epithelium at 7–14 daas. It then passes through the basal lamina to the visceral muscles and the hemocoel at 14–21 daas (Fig. 6, i–ii). Finally, it arrives at the salivary glands, where it invades type III cells and accumulates at 21–28 daas (Fig. 6, ii–iii). Although consideration should be given to the influence of insect stage and temperature, which can change the latency period between phytoplasma acquisition and transmission^34,35^, the infection dynamics determined in this study are consistent with those of previous studies in terms of the temporal localizations of OY and other phytoplasmas^14–16,24,36^ and spiroplasmas^8^. Thus, the proposed model may contribute to a better understanding of the detailed dynamics of plant pathogenic bacteria in their insect vectors.

The alimentary canal and salivary glands are the major barriers to the transmission of phytoplasmas^4,5,22,37^. Our results showed that the major site of OY phytoplasma infection in the alimentary canal is the anterior midgut (Table 1). In leafhopper, the anterior midgut extends throughout most of the abdomen^29^, and its large surface area facilitates the adsorption of phytoplasmas. A recent study showed that fluorescent beads coated with the membrane protein of FD phytoplasma mainly attached near the microvilli of gut epithelial cells in the anterior midgut of the vector *Euscelidius variegatus*^38^. Thus, the anterior midgut may contain an abundance of proteins that act as phytoplasma-binding receptors.

Gut epithelial cells are presumed to act as both a gut infection barrier^36^ and a gut escape barrier^39^. In the present study, OY phytoplasmas entered the epithelial cells of the anterior midgut by 7 daas and moved to the visceral muscles by 21 daas (Table 1, Fig. 3C–E, Supplementary Videos S1–3). These results imply that OY phytoplasmas pass through the gut infection barrier by 7 daas and the gut escape barrier by 21 daas (Fig. 6B).

Whole-mount immunofluorescence analysis revealed the localization of OY phytoplasmas in the visceral muscles of the alimentary canal (Figs. 3D–G and 5A–E), in agreement with our previous findings^27^. Electron and immunofluorescence microscopy analyses have revealed similar findings in spiroplasmas^6–8^ and liberibacter^40,41^. Thus, the visceral muscles of the alimentary canal appear to be a common infection site for insect-borne plant pathogenic bacteria that are transmitted in a persistent propagative manner.

Temporal microscopic observations of the visceral muscles (Table 1, Fig. 3D–G, Supplementary Videos S2–S4) revealed the presence of OY phytoplasmas in the internal circular muscle fibers and external longitudinal muscle fibers, as well as the extensive spread along these fibers (Fig. 6B). Previous studies have shown that Amp of OY phytoplasma and chrysanthemum yellows (CY) phytoplasma binds to insect microfilaments (including actin); this interaction is an important determinant of insect transmissibility^27,42^. Actin filaments, as the major component of muscle fibers, mediate the spread of phytoplasmas along the muscle fibers. In addition, OY phytoplasma was found along cytoplasmic actin in the gut epithelium (Supplementary Fig. S1E, Supplementary Video S3). Thus, both actin-based muscle fibers and cytoplasmic actin might be involved in the actin-based movement of OY phytoplasmas. Furthermore, actin is known to participate in the movement of insect-borne plant viruses^32,43,44^. We speculate that actin-based muscle fibers, which are required for plant virus movement, might also be needed for phytoplasma movement.

The transmission of phytoplasmas requires passage through the salivary glands of their insect vectors. Our study showed that the major site of OY phytoplasma infection in salivary glands was type III cells (Table 1). In leafhopper, type III cells are the largest cells and contain many intracellular canaliculi^31^, which also facilitate the adsorption and excretion of phytoplasmas. The infection and attachment of FD phytoplasmas to type III cells of the vector *E. variegatus* have also been reported^38,45,46^. These findings suggest the existence of phytoplasma-binding proteins that act as receptors on type III cells.

Salivary gland cells are presumed to act as a salivary gland infection barrier^47^. In this study, OY phytoplasmas accumulated on the cell surfaces of type III cells by 21 daas, and entered the cells by 28 daas (Table 1, Fig. 4C–F, Supplementary Videos S5–S7), indicating that OY phytoplasmas pass through the salivary gland infection barrier at 21–28 daas. For transmission to plants, phytoplasmas must pass through the apical plasmalemma, which presumably acts as a salivary gland escape barrier^5,48^. After the invasion of salivary gland cells, phytoplasmas may enter apical plasmalemma-lined cavities, then enter the salivary duct. Previous reports strongly support this hypothesis—electron microscopy studies showed that clover phyllody phytoplasmas were located near and inside the salivary gland cavities of its vector, *Euscelis lineolatus*^14,49^. Our study revealed the occasional presence of OY phytoplasmas in the space near the apical plasmalemma at 28 daas (Fig. 4F). Considering that OY phytoplasmas were transmitted by *M. striifrons* to new plants within approximately 24–25 days at 25 °C^50^, we speculate that OY phytoplasmas pass through the apical plasmalemma (i.e., salivary gland escape barrier) into the salivary duct by 28 daas (Fig. 6C).

Invasion of the salivary glands is a necessary step for successful insect transmission of phytoplasmas. A previous study regarding CY phytoplasma in the heads of the vector *E. variegatus* demonstrated a correlation between lack of transmission and low accumulation of the pathogen^22^. Our study indicated that phytoplasma invasion into type III cells occurred after sufficient accumulation of the pathogen on the surfaces of salivary gland cells (Figs. 2B, 4C–F). Therefore, this step plays a key role in both the efficient invasion of salivary glands and further successful transmission of phytoplasmas to new plants.

The immunohistochemistry-based localization of OY phytoplasmas in the brain (Fig. 1) is consistent with the findings of previous studies, which also confirmed the presence of phytoplasmas in the brain by TEM and ELISA^15,17,51^. Although the effects of phytoplasma infection in the brain are unknown, it would be interesting to analyze whether the infection can change the behavior of insect vectors in a manner beneficial to phytoplasmas.

In this study, we elucidated the overall infection dynamics of OY phytoplasma in *M. striifrons*, especially in the alimentary canal and salivary glands. Although the mechanisms of phytoplasma passage through the barriers, the alimentary canal and salivary glands, still remains unclear, possible mechanisms of movement through the barriers have been suggested from cultivable spiroplasmas^52^. Spiroplasmas are thought to adhere to receptors on the cell membrane, be taken into the cytoplasm by endocytosis, and released by exocytosis. Phytoplasmas are also thought to adhere to cells of insect vectors, and recent studies have shown that several membrane proteins are important for phytoplasma transmission, such as Amp, ORF3, and P38 of OY phytoplasma^26,27,53^, Amp of CY phytoplasma^42,54^, and variable membrane proteins of FD phytoplasma^38^. However, the functions of these membrane proteins are still elusive. Further analyses of these proteins are needed to determine their stage-specific involvements in phytoplasma infection. This information would provide insight into the molecular mechanisms of phytoplasma transmission by their insect vectors.

## Methods

### Insect and phytoplasma growth conditions

OY phytoplasma was originally obtained in Saga Prefecture, Japan^55^ and has been maintained on garland chrysanthemum (*Chrysanthemum coronarium*) using a leafhopper vector (*Macrosteles striifrons*) in growth chambers maintained at 25 °C under a 16-h-light/8-h-dark cycle. In all experiments involving temporal analysis of OY phytoplasma-infected leafhoppers, third- or fourth-instar nymphs of non-infected leafhoppers were allowed a 7-day acquisition feeding period on an OY phytoplasma-infected garland chrysanthemum, then transferred to a healthy garland chrysanthemum. At different daas intervals, insects were collected for experiments. Leafhoppers feeding on diseased plants and healthy plants for more than 1 month were used as positive (OY-infected) and negative (non-infected) controls, respectively.

### Immunohistochemical staining-based 3D imaging

At 1, 2, 4, 6, 10, 14, 20, 27, 34, and 41 daas, four leafhoppers were collected and fixed overnight in 4% paraformaldehyde. The fixed samples were dehydrated in an ethanol dilution series and embedded in Paraplast Plus (Sherwood Medical, St. Louis, MO, USA). The embedded samples were serially cut into 10-µm sections using a microtome (PR-50; Yamato Kohki, Saitama, Japan), resulting in 40–60 sections per insect. The sections were deparaffinized, rehydrated, and processed for immunohistochemical staining, as described previously^56^. OY phytoplasmas were detected using anti-Amp IgG and an alkaline-phosphatase-mediated reporter system, as described previously^26^. Immunohistochemically stained sections were observed and imaged by epifluorescence microscopy (BX60F-3; Olympus, Tokyo, Japan). All images of serial sections from the entire insect embedding block were processed and aligned using characteristic organs (i.e., eyes); 3D images were reconstructed using TRI 3D SRF II software (RATOC System Engineering Co., Ltd., Tokyo, Japan).

### Phytoplasma titer quantification by qPCR

At 7, 14, 21, and 28 daas, at least eight leafhoppers (whole body-level quantification) and up to 12 leafhoppers (organ- or tissue-level quantification) were collected and processed for experiments. For whole body-level quantification, total genomic DNA was extracted from each intact insect using the DNeasy Blood and Tissue Kit (QIAGEN, Hilden, Germany), in accordance with the manufacturer’s instructions. For organ- or tissue-level quantification, internal organs (i.e., alimentary canal and salivary glands) and legs (representative of the hemocoel) were dissected in phosphate-buffered saline (PBS). Four organs/tissues were treated as one sample to obtain a sufficient amount of DNA. Total genomic DNA was extracted using the cetyltrimethylammonium bromide method^57^.

The phytoplasma titer was quantified in a qPCR assay using the Thermal Cycler Dice real-time PCR system (TaKaRa, Shiga, Japan) with SYBR Premix ExTaq (TaKaRa), as described previously^58^. A primer set for the OY gene encoding the elongation factor Tu (tufB) was used for phytoplasma detection^59^. As an internal control, the 18S rRNA gene of the leafhopper host *M. striifrons* was amplified using Ms18SF (5′-AACACGGGAAACCTCACC-3′) and Ms18SR (5′-CAGACAAATCGCTCCACCAA-3′) primers. The relative value of tufB gene compared to 18S rRNA gene for each sample was obtained by two technical replicates; the relative phytoplasma titer (tufB gene) was normalized relative to the 18S rRNA gene in each sample. All experiments were performed at least three times and OY-phytoplasma-positive samples of all experiments were used to create graphs.

### Whole-mount immunofluorescence staining

Seven or more leafhoppers were collected at 7, 14, 21, and 28 daas; their internal organs were then dissected. Immunofluorescence staining was performed in accordance with the procedure previously described by Wei *et al*.^60^, with some modifications. The dissected organs were quickly immersed in fixative (4% paraformaldehyde in 0.1% Triton X-100 in PBS [PBT], pH 7.4) and incubated overnight at 4°C, as described previously^9^. After samples had been washed with PBS, they were permeabilized in blocking buffer (1% bovine serum albumin in PBT) for 30 min at room temperature. The samples were washed again with PBS, then incubated for 1.0–1.5 h at 37 °C with PBT containing Alexa Fluor 488 (Invitrogen, Carlsbad, CA, USA) directly conjugated to anti-Amp IgG (Amp-Alexa Fluor 488). The samples were subsequently washed twice with PBT, post-fixed in fixative solution for 15 min at room temperature, washed with PBS, and stained with phalloidin-Alexa Fluor 546 (Invitrogen) for 20 min at room temperature to detect leafhopper actin. After an additional PBS wash, the samples were mounted on glass slides with ProLong Gold antifade reagent (Invitrogen) and visualized under a TCS SP5 confocal laser scanning microscope (Leica, Wetzlar, Germany). For Z-stack analysis, 50–100 images of z-sections were obtained at 0.5-µm intervals, except for the 0.2-µm intervals shown in Fig. 3G. All experiments were performed at least three times.

### Statistical analysis

The data were analyzed using R (version 3.5.0; R Foundation for Statistical Computing, Vienna, Austria). The relative phytoplasma titer was log-transformed before statistical analysis. Multiple comparisons among means were performed using Tukey’s honestly significant difference test, with differences considered statistically significant when *p* <0.05. The data were back-transformed for presentation.

## Supplementary information

Supplementary Information.

Supplementary Information 2.

Supplementary Information 3.

Supplementary Information 4.

Supplementary Information 5.

Supplementary Information 6.

Supplementary Information 7.

Supplementary Information 8.

## Data Availability

All data are available upon request.

## References

[1] Chen Q, Wei T (2016). Viral receptors of the gut: insect-borne propagative plant viruses of agricultural importance. Curr. Opin. Insect Sci..

[2] Perilla-Henao LM, Casteel CL (2016). Vector-borne bacterial plant pathogens: interactions with hemipteran insects and plants. Front. Plant Sci..

[3] Hogenhout SA, Ammar ED, Whitfield AE, Redinbaugh MG (2008). Insect vector interactions with persistently transmitted viruses. Annu. Rev. Phytopathol..

[4] Hogenhout S (2008). Phytoplasmas: bacteria that manipulate plants and insects. Mol. Plant Pathol..

[5] Weintraub PG, Beanland L (2006). Insect vectors of phytoplasmas. Annu. Rev. Entomol..

[6] Kwon MO, Wayadande AC, Fletcher J (1999). *Spiroplasma citri* movement into the intestines and salivary glands of its leafhopper vector, *Circulifer tenellus*. Phytopathology.

[7] Özbek E, Miller SA, Meulia T, Hogenhout SA (2003). Infection and replication sites of *Spiroplasma kunkelii* (Class: Mollicutes) in midgut and Malpighian tubules of the leafhopper *Dalbulus maidis*. J. Invertebr. Pathol..

[8] Liu HY, Gumpf DJ, Oldfield GN, Calavan EC (1983). The relationship of *Spiroplasma citri* and *Circulifer tenellus*. Phytopathology.

[9] Ammar ED, Hogenhout SA (2005). Use of immunofluorescence confocal laser scanning microscopy to study distribution of the bacterium corn stunt spiroplasma in vector leafhoppers (Hemiptera: Cicadellidae) and in host plants. Ann. Entomol. Soc. Am..

[10] Ammar ED, Shatters RG, Hall DG (2011). Localization of *Candidatus* Liberibacter asiaticus, associated with citrus huanglongbing disease, in its psyllid vector using fluorescence *in situ* hybridization. J. Phytopathol..

[11] Gasparich GE (2010). Spiroplasmas and phytoplasmas: microbes associated with plant hosts. Biologicals.

[12] Namba S (2019). Molecular and biological properties of phytoplasmas. Proc. Jpn. Acad. Ser. B Phys. Biol. Sci..

[13] Sinha RC, Chiykowski LN (1967). Initial and subsequent sites of aster yellows virus infection in a leafhopper vector. Virology.

[14] Gouranton J, Maillet PL (1973). High resolution autoradiography of mycoplasmalike organisms multiplying in some tissues of an insect vector for clover-phyllody. J. Invertebr. Pathol..

[15] Nasu S, Jensen DD, Richardson J (1970). Electron microscopy of mycoplasma-like bodies associated with insect and plant hosts of peach western X-disease. Virology.

[16] Sinha RC, Paliwal YC (1970). Localization of a Mycoplasma-like organism in tissues of a leafhopper vector carrying clover phyllody agent. Virology.

[17] Lefol C (1994). Propagation of Flavescence dorée MLO (mycoplasma-like organism) in the leafhopper vector *Euscelidius variegatus* Kbm. J. Invertebr. Pathol..

[18] Nakajima S (2009). Movement of onion yellows phytoplasma and *Cryptotaenia japonica* witches’ broom phytoplasma in the nonvector insect *Nephotettix cincticeps*. Jpn. J. Phytopathol..

[19] De Oliveira E, Santos JC, Magalhães PC, Cruz I (2007). Maize bushy stunt phytoplasma transmission by *Dalbulus maidis* is affected by spiroplasma acquisition and environmental conditions. Bull. Insectol..

[20] Rashidi M, D’amelio R, Galetto L, Marzachì C, Bosco D (2014). Interactive transmission of two phytoplasmas by the vector insect. Ann. Appl. Biol..

[21] Bosco D (2007). Interrelationships between “*Candidatus* Phytoplasma asteris” and its leafhopper vectors (Homoptera: Cicadellidae). J. Econ. Entomol..

[22] Galetto L (2009). Variation in vector competency depends on chrysanthemum yellows phytoplasma distribution within *Euscelidius variegatu*s. Entomol. Exp. Appl..

[23] Pacifico D (2015). Decreasing global transcript levels over time suggest that phytoplasma cells enter stationary phase during plant and insect colonization. Appl. Environ. Microbiol..

[24] Roddee J, Kobori Y, Hanboonsong Y (2019). Characteristics of sugarcane white leaf phytoplasma transmission by the leafhopper *Matsumuratettix hiroglyphicus*. Entomol. Exp. Appl..

[25] Kakizawa S (2004). Secretion of immunodominant membrane protein from onion yellows phytoplasma through the Sec protein-translocation system in *Escherichia coli*. Microbiology.

[26] Ishii Y (2009). In the non-insect-transmissible line of onion yellows phytoplasma (OY-NIM), the plasmid-encoded transmembrane protein ORF3 lacks the major promoter region. Microbiology.

[27] Suzuki S (2006). Interaction between the membrane protein of a pathogen and insect microfilament complex determines insect-vector specificity. Proc. Natl. Acad. Sci. USA.

[28] Berlin LC, Hibbs ET (1963). Digestive system morphology and salivary enzymes of the potato leafhopper, *Empoasca fabae* (Harris). Proc. Iowa Acad. Sci..

[29] Tsai JH, Perrier JL (1996). Morphology of the digestive and reproductive systems of *Dalbulus maidis* and *Graminella nigrifrons* (Homoptera: Cicadellidae). Fla. Entomol..

[30] Grubaugh ND (2016). Genetic drift during systemic arbovirus infection of mosquito vectors leads to decreased relative fitness during host switching. Cell Host Microbe.

[31] Sogawa K (1965). Studies on the salivary glands of rice plant leafhoppers. Jpn. J. Appl. Entomol. Zool..

[32] Chen Q (2012). Tubular structure induced by a plant virus facilitates viral spread in its vector insect. PLoS Pathog..

[33] Mao Q (2017). Filamentous structures induced by a phytoreovirus mediate viral release from salivary glands in its insect vector. J. Virol..

[34] Alma A (2018). New insights in phytoplasma-vector interaction: acquisition and inoculation of flavescence dorée phytoplasma by *Scaphoideus titanus* adults in a short window of time. Ann. Appl. Biol..

[35] Murral DJ, Nault LR, Hoy CW, Madden LV, Miller SA (1996). Effects of temperature and vector age on transmission of two Ohio strains of aster yellows phytoplasma by the aster leafhopper (Homoptera: Cicadellidae). J. Econ. Entomol..

[36] Nakajima S (2002). Detection of mulberry dwarf and onion yellows phytoplasmas by PCR from vector insects and nonvector insects. Jpn. J. Phytopathol..

[37] Bosco, D., & D’Amelio, R. Transmission specificity and competition of multiple phytoplasmas in the insect vector. In: *Phytoplasmas: genomes, plant hosts and vectors* (ed. Weintraub, P. G., & Jones, P.) 293–308 (CAB International, 2010).

[38] Arricau-Bouvery N (2018). Variable membrane protein A of flavescence dorée phytoplasma binds the midgut perimicrovillar membrane of *Euscelidius variegatus* and promotes adhesion to its epithelial cells. Appl. Environ. Microbiol..

[39] Sinha RC, Chiykowski LN (1967). Multiplication of aster yellows virus in a nonvector leafhopper. Virology.

[40] Kruse A (2017). Combining’omics and microscopy to visualize interactions between the Asian citrus psyllid vector and the Huanglongbing pathogen *Candidatus* Liberibacter asiaticus in the insect gut. PLoS One.

[41] Mann M (2018). *Diaphorina citri* nymphs are resistant to morphological changes induced by “*Candidatus* Liberibacter asiaticus” in midgut epithelial cells. Infect. Immun..

[42] Galetto L (2011). The major antigenic membrane protein of “*Candidatus* Phytoplasma asteris” selectively interacts with ATP synthase and actin of leafhopper vectors. PLoS One.

[43] Wei T (2006). The spread of *Rice dwarf viru*s among cells of its insect vector exploits virus-induced tubular structures. J. Virol..

[44] Jia D (2014). Virus-induced tubule: a vehicle for rapid spread of virions through basal lamina from midgut epithelium in the insect vector. J. Virol..

[45] Lherminier J, Prensier G, Boudon-Padieu E, Caudwell A (1990). Immunolabeling of grapevine flavescence dorée MLO in salivary glands of *Euscelidius variegatus*: a light and electron microscopy study. J. Histochem. Cytochem..

[46] Lefol C, Caudwell A, Lherminier J, Larrue J (1993). Attachment of the flavescence dorée pathogen (MLO) to leafhopper vectors and other insects. Ann. Appl. Biol..

[47] Purcell AH, Richardson J, Finlay A (1981). Multiplication of the agent of X‐disease in a non‐vector leafhopper *Macrosteles fascifrons*. Ann. Appl. Biol..

[48] Wayadande AC, Baker GR, Fletcher J (1997). Comparative ultrastructure of the salivary glands of two phytopathogen vectors, the beet leafhopper, *Circulifer tenellus* (Baker), and the corn leafhopper, *Dalbulus maidis* Delong and Wolcott (Homoptera: Cicadellidae). Int. J. Insect Morphol. Embryol..

[49] Gourret JP, Maillet PL, Gouranton J (1973). Virus-like particles associated with the mycoplasmas of clover phyllody in the plant and in the insect vector. Microbiology.

[50] Shiomi T (1998). A symptomatic mutant of onion yellows phytoplasma derived from a greenhouse-maintained isolate. Ann. Phytopathol. Soc. Jpn..

[51] Kawakita H (2000). Identification of mulberry dwarf phytoplasmas in the genital organs and eggs of leafhopper *Hishimonoides sellatiformis*. Phytopathology.

[52] Fletcher J, Wayadande A, Melcher U, Ye F (1998). The phytopathogenic mollicute-insect vector interface: a closer look. Phytopathology.

[53] Neriya Y (2014). Onion yellow phytoplasma P38 protein plays a role in adhesion to the hosts. FEMS Microbiol. Lett..

[54] Rashidi M (2015). Role of the major antigenic membrane protein in phytoplasma transmission by two insect vector species. BMC Microbiol..

[55] Miyahara K, Matsuzaki M, Tanaka K, Sako N (1982). A new disease of onion caused by mycoplasma-like organism in Japan. Ann. Phytopathol. Soc. Jpn..

[56] Oshima K (2001). Isolation and characterization of derivative lines of the onion yellows phytoplasma that do not cause stunting or phloem hyperplasia. Phytopathology.

[57] Namba S (1993). Detection and differentiation of plant-pathogenic mycoplasmalike organisms using polymerase chain reaction. Phytopathology.

[58] Himeno M (2014). Purple top symptoms are associated with reduction of leaf cell death in phytoplasma-infected plants. Sci. Rep..

[59] Oshima K (2011). Dramatic transcriptional changes in an intracellular parasite enable host switching between plant and insect. PLoS One.

[60] Wei T (2006). Pns12 protein of *Rice dwarf virus* is essential for formation of viroplasms and nucleation of viral-assembly complexes. J. Gen. Virol..

