# Supplementary material for: Spatiotemporal dynamics and quantitative analysis of phytoplasmas in insect vectors
Source: Sci Rep. 2020 Mar 9;10:4291. doi: 10.1038/s41598-020-61042-x (PMC7062745; doi:10.1038/s41598-020-61042-x)
Supplement: Supplementary file 8 — Supplementary Information 8. [file 41598_2020_61042_MOESM8_ESM.pdf]

Supplementary Information

Spatiotemporal dynamics and quantitative analysis of phytoplasmas in insect vectors

Hiroaki Koinuma[1], Kensaku Maejima[1], Ryosuke Tokuda[1], Yugo Kitazawa[1], Takamichi Nijo[1], Wei Wei[2], Kohei Kumita[1], Akio Miyazaki[1], Shigetou Namba[1], and Yasuyuki Yamaji[1]*

[1] Graduate School of Agricultural and Life Sciences, The University of Tokyo, 1-1-1 Yayoi, Bunkyo-ku, Tokyo 113-8657, Japan.
[2] Molecular Plant Pathology Laboratory, US Department of Agriculture–Agricultural Research Service, Beltsville, MD 20705

*Corresponding author
Address: Graduate School of Agricultural and Life Sciences, The University of Tokyo, 1-1-1 Yayoi, Bunkyo-ku, Tokyo 113-8657, Japan.
Tel: +81-3-5841-5092.
Fax: +81-3-5841-5090.
E-mail: ayyamaji@mail.ecc.u-tokyo.ac.jp

Supplementary Table S1. Detection of OY phytoplasma in *M. striifrons* by qPCR

No. of daas[b]	No. of positive insects or dissected tissues[a] detected by qPCR at different daas							
	entire insect body		alimentary canal		hemocoel[c]		salivary glands	
	No. detected / no. tested	ratio (%)	No. detected / no. tested	ratio (%)	No. detected / no. tested	ratio (%)	No. detected / no. tested	ratio (%)
7	25/27	93	12/12	100	9/12	75	6/12	50
14	13/22	59	12/12	100	11/12	92	9/12	75
21	15/23	65	11/12	92	12/12	100	9/12	75
28	19/23	83	10/10	100	10/10	100	10/10	100

[a] Four dissected tissues were prepared as one sample.

[b] daas: days after acquisition start on the diseased plants

[c] Legs were used as a representative of the hemocoel.

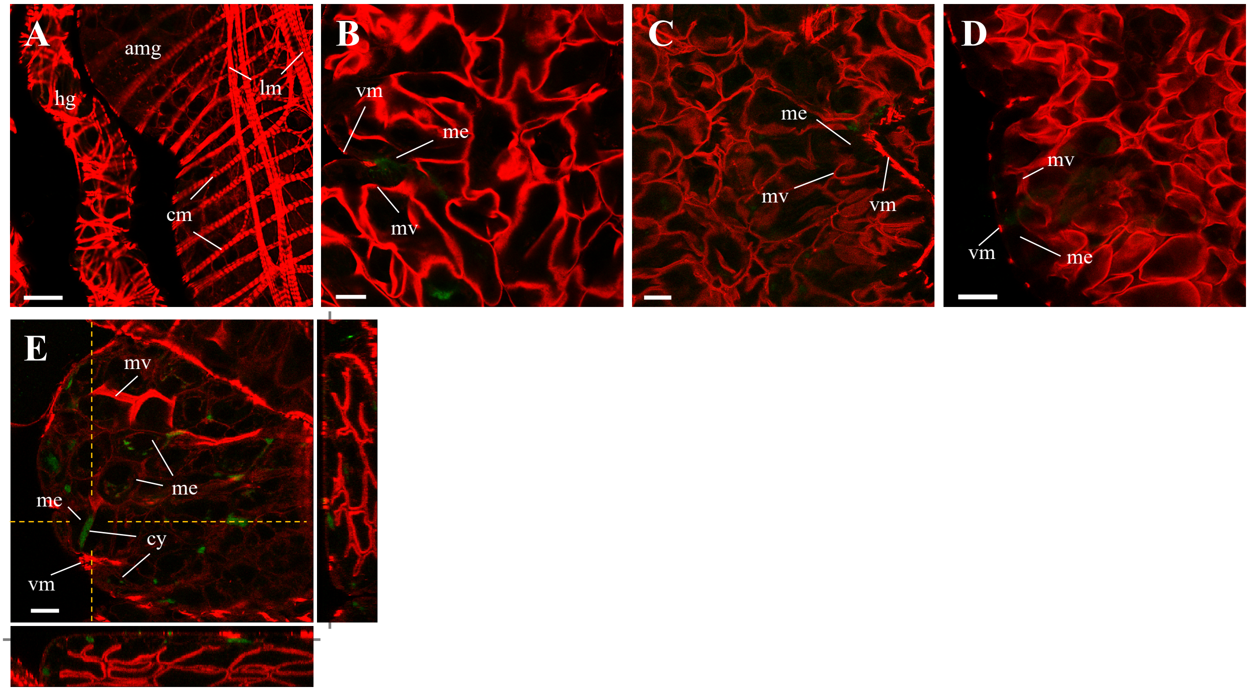

Supplementary Figure S1. Distribution of OY phytoplasma in the alimentary canal of *M. striifrons*, supplementary to Fig. 3C–F.

Whole-mount immunofluorescence staining of the alimentary canal using Amp-Alexa Fluor 488 (green) and the actin dye phalloidin-Alexa Fluor 546 (red). Muscle side of anterior midguts of OY-infected leafhoppers at 7 days after acquisition start (daas) (A) and luminal side at 14 daas (B), 21 daas (C, E), or 28 daas (D). Panels A–D represent the same samples as in the image a of Fig. 3C–F. Panel E represents the same sample as in the image b of Fig. 3E. Images in panel E were obtained as described in Fig. 3. amg, anterior midgut; hg, hindgut; cm, circular muscle; lm, longitudinal muscle; me, midgut epithelium; mv, microvilli; vm, visceral muscle; cy, cytoplasmic actin. Bar, 25 µm.

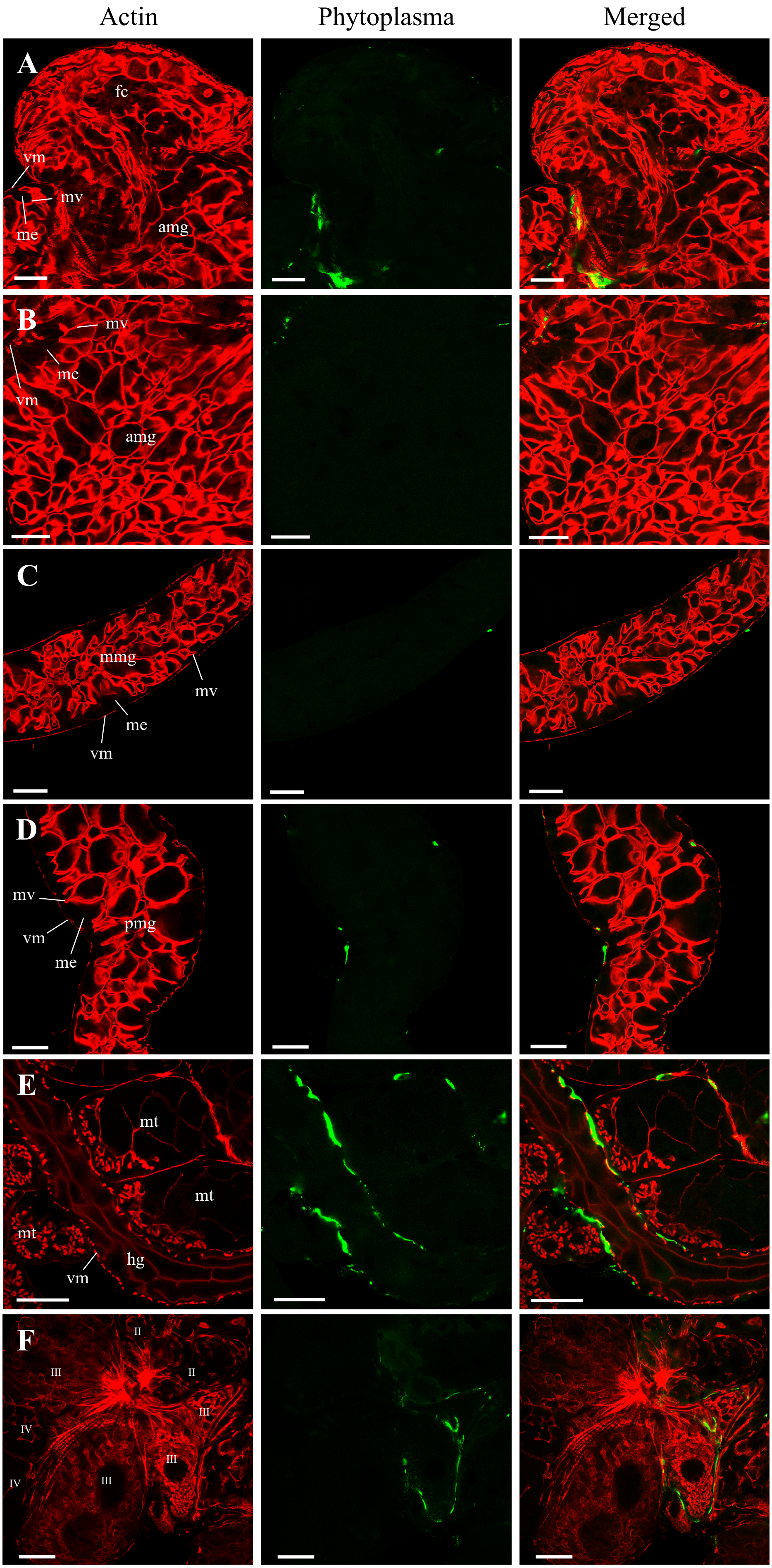
Actin
Phytoplasma
Merged
A
fc
vm
mv
me
amg
B
mv
me
vm
amg
C
mmg
mv
me
vm
D
mv
vm
pmg
me
E
mt
mt
mt
hg
vm
F
II
III
II
III
IV
III
IV
III

Supplementary Figure S2. Distributions of OY phytoplasma in the internal organs or tissues of *M. striifrons*, supplementary to Fig. 5.

Whole-mount immunofluorescence staining of the internal organs of OY-infected leafhoppers at 28 days after acquisition start (daas) using Amp-Alexa Fluor 488 (green) and the actin dye phalloidin-Alexa Fluor 546 (red). Luminal side of the filter chamber (fc) (A), anterior midgut (amg) (B), middle midgut (mmg) (C), posterior midgut (pmg) (D), and hindgut (hg) and Malpighian tubules (mt), (E) and the inner side of the salivary gland (F). Panels A–F represent the same samples as in Fig. 5A–F. Images are representative of more than three experiments. me, midgut epithelium; mv, microvilli; vm, visceral muscle; (II)–(IV), type II–IV cells. Bar, 50 µm.

Supplementary Video S1. Z-stack video showing the localization of OY phytoplasmas in the alimentary canal at 7 days after acquisition start (daas) , corresponding to panel b in Fig. 3C
Z-stack images were obtained at 0.5 μm intervals. All images were merged with green fluorescence (phytoplasma) and red fluorescence (actin).

Supplementary Video S2. Z-stack video showing the localization of OY phytoplasmas in the alimentary canal at 14 daas, corresponding to panel b in Fig. 3D
Z-stack images were obtained at 0.5 μm intervals. All images were merged with green fluorescence (phytoplasma) and red fluorescence (actin).

Supplementary Video S3. Z-stack video showing the localization of OY phytoplasmas in the alimentary canal at 21 daas, corresponding to panel b in Fig. 3E
Z-stack images were obtained at 0.5 μm intervals. All images were merged with green fluorescence (phytoplasma) and red fluorescence (actin).

Supplementary Video S4. Z-stack video showing the localization of OY phytoplasmas in the alimentary canal at 28 daas, corresponding to panel b in Fig. 3F
Z-stack images were obtained at 0.5 μm intervals. All images were merged with green fluorescence (phytoplasma) and red fluorescence (actin).

Supplementary Video S5. Z-stack video showing the localization of OY phytoplasmas in the salivary glands at 14 daas, corresponding to panel b in Fig. 4C
Z-stack images were obtained at 0.5 μm intervals. All images were merged with green fluorescence (phytoplasma) and red fluorescence (actin).

Supplementary Video S6. Z-stack video showing the localization of OY phytoplasmas in the salivary glands at 21 daas, corresponding to panel b in Fig. 4D
Z-stack images were obtained at 0.5 μm intervals. All images were merged with green fluorescence (phytoplasma) and red fluorescence (actin).

Supplementary Video S7. Z-stack video showing the localization of OY phytoplasmas in the salivary glands at 28 daas, corresponding to panel b in Fig. 4E
Z-stack images were obtained at 0.5 μm intervals. All images were merged with green fluorescence (phytoplasma) and red fluorescence (actin).
